# Associations of Pre-Defined Dietary Patterns with Obesity Associated Phenotypes in Tehranian Adolescents

**DOI:** 10.3390/nu8080505

**Published:** 2016-08-18

**Authors:** Sahar Mohseni-Takalloo, Firoozeh Hosseini-Esfahani, Parvin Mirmiran, Fereidoun Azizi

**Affiliations:** 1 Faculty of Medicine, Bam University of Medical Sciences, 7661771967 Bam, Iran; s.mohseni@mubam.ac.ir; 2 Nutrition and Endocrine Research Center, Research Institute for Endocrine Sciences, Shahid Beheshti University of Medical Sciences, P.O. Box 19395-4763, 1985717413 Tehran, Iran; f.hosseini@sbmu.ac.ir; 3 Endocrine Research Center, Research Institute for Endocrine Sciences, Shahid Beheshti University of Medical Sciences, 1985717413 Tehran, Iran; azizi@endocrine.ac.ir

**Keywords:** adolescent, DGAI, HEI-2005, HEI-2010, obesity

## Abstract

Obesity has become a public health problem in adolescents and could be a risk factor for both short-term and long-term health consequences. This study aimed to evaluate the relationship of Dietary Guidelines for Americans Adherence Index (DGAI), Healthy Eating Index-2005 (HEI-2005) and Healthy Eating Index-2010 (HEI-2010) with risk of obesity associated phenotypes in Tehranian adolescents. This cross-sectional study was conducted within the framework of the Tehran Lipid and Glucose Study, on 722 adolescents, aged 10–19 years. Usual dietary intakes were assessed by a food frequency questionnaire, and diet quality scores were obtained based on DGAI, HEI-2005 and HEI-2010. General obesity and cardio metabolic risk factors were defined according to the Centers of Disease Control and Prevention and de’Ferranti cut-offs, respectively. After adjusting for age, sex, energy intake and physical activity, compared to those in the lowest quartile, participants in the highest quartile of HEI-2010 had lower risk of general obesity (OR: 0.62; 95% CI: 0.38–0.93; *P*_trend_ = 0.03) and central obesity (OR: 0.63; 95% CI: 0.44–0.95; *P*_trend_ = 0.04). No association was observed between different types of obesity and scores of other diet quality indices. In conclusions, considering the role of HEI-2010 in decreasing the risk of obesity, these findings suggest that HEI-2010 may be useful for assessing diet-related progress in obesity prevention efforts.

## 1. Introduction

Obesity has become a public health problem worldwide [1] and adolescence is a vulnerable period for the development of obesity [2]. In recent decades, increase in the prevalence of adolescent obesity has been reported in many developing countries [3,4]. According to national cutoffs, the prevalence of obesity among Iranian adolescents was 4.8% in 2003–2004, which rose to 15% in 2008–2011 [5]. In adolescents, obesity could be a risk factor for both short- and long-term health consequences [6], which include metabolic syndrome, cardiovascular disease and diabetes at later ages [7]. However, not all obese adolescents exhibit these cardio-metabolic phenotypes. Evidence shows that metabolically healthy obese (MHO) individuals have lower incidence of cardiovascular disease and type II diabetes when compared to their metabolically unhealthy obese (MUO) counterparts [8].

Among risk factors of obesity, special attention is paid to dietary patterns, as a controllable environmental factor [9]. Different foods are used together and interactions between nutrients are possible [10]. The examination of overall diet quality has been used in order to reflect the complexity of food intake patterns and dietary exposure. Two methods are used to evaluate dietary patterns: (1) dietary patterns based on statistical modeling of available dietary data, like cluster analysis and factor analysis; (2) predetermined dietary patterns based on previous knowledge of a healthy diet, like indices of diet quality [11]. The US Departments of Agriculture and Health and Human Services have issued dietary recommendations, the dietary guidelines for Americans (DGA), to help reduce the risk of cardiovascular disease and other chronic diseases [12]. Dietary guidelines for Americans adherence index (DGAI), healthy eating index 2005 (HEI-2005) and healthy eating index 2010 (HEI-2010) are three indices for evaluating diet quality; the first two indices (DGAI and HEI-2005) were developed according to the sixth version of DGA (DGA-2005) and HEI-2010 was developed according to the seventh version of the DGA (DGA-2010). DGA-2005 was published with new concepts of diet, emphasizing the important aspects of diet quality, such as whole grain, various types of vegetables and specific types of fat, and introducing the new concept of “discretionary calories” [11]. Some features of DGA-2005 have been changed in DGA-2010; renaming the meat-beans and milk groups to “protein foods” and “dairy groups”, respectively; vegetable subgroups now provide more achievable intake recommendations, increased amounts of sea foods are recommended, and a limit on calories from solid fats and added sugars is included [13]. 

Standard quantitative dietary guidelines are not available in Iran, although evidently the use of these indices can be beneficial for nutritional assessment. In previous studies the association between diet quality indices and obesity has been investigated in adolescents. It had been shown that an increased HEI-1995 score was not associated with waist circumference (WC), but was negatively associated with BMI [14]. In addition, no significant relationship was found between Mediterranean diet score (MDS) and WC [15]. Also, MHO adolescents had higher total HEI-2005 scores compared with their MUO counterparts [8]. Comparing results from these studies is not easy because of differences in diet quality indices, study populations and statistical analysis methods.

To our knowledge, no study has hither examined adherence to multiple indices based on DGA recommendations in an adolescent population or to determine which index can better demonstrate the risk of obesity associated phenotypes. Thus, the aim of the present study was (1) to evaluate adherence of Tehranian adolescents to DGAI, HEI-2005 and HEI-2010; (2) to assess the relationship between each of these indices with obesity and obesity associated phenotypes and (3) to determine whether HEI-2010, compared to 2005 indices, demonstrates the relationship with obesity-associated phenotypes better, a comparison which would provide valuable comparative data on how existing diet quality indices relate to obesity status in adolescents.

## 2. Methods and Materials

### 2.1. Population and Sampling

This cross-sectional study was conducted within the framework of Tehran Lipid and Glucose Study (TLGS, an ongoing prospective population based investigation being performed in residents of district No. 13 of Tehran, the capital of Iran, to detect and prevent non-communicable diseases and their risk factors [16,17]. 

During the fourth phase of TLGS (2008–2011), of 1454 adolescents (aged 10–19 years) who completed the examination, 848 were randomly selected for completing the dietary assessment. Participants were excluded from the study if they did not have complete anthropometric or physical activity data (*n* = 4), had special diets (*n* = 16), received any thyroid medication (*n* = 9) or reported energy intake ≤800 kcal or ≥5000 kcal for boys and ≤600 kcal or ≥4500 kcal for girls (*n* = 97) [18]. Finally, the data for 722 adolescents (392 girls and 330 boys) were analyzed.

Written informed consent was obtained from all those aged over 18 years and from the parents of those aged <18 years; the study protocol was approved by the ethics committee and research council of the Research Institute for Endocrine Science, Shahid Beheshti University of Medical Sciences.

### 2.2. Dietary Intake Assessment

Usual dietary data were collected using a validated semi-quantitative food frequency questionnaire (FFQ), which contained 168 food items [19,20]. Trained dietitians completed the FFQ during face-to-face interviews, asking participants to report their frequency of consumption of each food item, during the past year on a daily, weekly or monthly basis. These reports were converted to daily intakes. The Food Composition Table (FCT) of the US Department of Agriculture (USDA) was used for analyzing nutrient intakes of each food and beverages [21], because the Iranian FCT is incomplete and provides limited data on nutrient contents of raw foods and beverages [22].

### 2.3. Diet Quality Scores

The DGAI score [23] has a total of 20 dietary items which assess adherence to the key dietary intake recommendations of DGA 2005 for the general public. Eleven items assessed the calorie-specific “food group recommendations”, and 9 items are related to “healthy choice recommendations”, the maximum score of each item being 1.0. Most items had a partial adherence score of 0.5, and 0 points were given when the recommendation was not achieved. DGAI considers a penalty of 0.5 points for overconsumption of energy-dense food groups, including meat, dairy, grains and starchy vegetables. Since alcohol consumption has been prohibited for adolescents in DGA-2005, and we were unable to measure the alcoholic beverage intake because alcohol consumption is prohibited in Iran and participants refrain from reporting their consumption, we excluded the “alcohol intake” item from this index while the other 19 items were examined.

The Healthy Eating Index-2005 (HEI-2005) [24] contains 12 food components that reflect the key recommendations of DGA-2005. The first six components (including total fruit, whole fruit, total vegetables, dark green and orange vegetables, legumes, total grain and whole grain) were scored from 0 to 5 points; the next five components (including milk, meat and beans, oil, saturated fat and sodium) were scored from 0 to 10 points; and the last component (percent of calorie intake from Solid fats, Alcoholic beverages, and Added Sugars = SoFAAS) was scored from 0 to 20 points. Scores were calculated proportionally, except for two components (saturated fat and sodium) which were prorated linearly between 0 to 8 and 8 to 10 points respectively (8 and 10 points demonstrated acceptable and optimal levels, respectively). The maximum possible score for HEI-2005 is 100 and higher scores indicate a better diet quality. In this index dietary intake recommendations are expressed per 1000 kcal energy intake for all components, except for saturated fat and SoFAAS.

Publication of the DGA-2010 necessitated an update to the HEI-2005 to capture key changes in this version of DGA; therefore, the HEI-2010 [25] was developed. Changes to this index include (a) greens and beans replaced with dark green, orange vegetables and legumes; (b) seafood and plant proteins (which represent selected subgroups of protein foods) have been added; (c) fatty acids (a ratio of polyunsaturated and monounsaturated to saturated fatty acids) replaced two components; saturated fat and oils and (d) refined grains, replaced the adequacy component, total grains, to assess over-consumption. HEI-2010, like the earlier version, has 12 food components; six components (including total fruit, whole fruit, total vegetable, greens and beans, total protein foods, sea food and plant proteins) were scored from 0 to 5 points; five components (including whole grains, dairy, fatty acids, refined grains and sodium) were scored from 0 to 10 points; and the last component (percent of calorie intake from solid fats, alcohol, and added sugars (empty calories)) was scored from 0 to 20 points. Intakes between the minimum and maximum standards are scored proportionate. It should be noted that the HEI-2010 considers alcohol above a threshold level (>13 g/1000 kcal), indicative of moderate drinking, as empty calories. In this index, similar to HEI-2005, most of dietary intake recommendations are expressed per 1000 kcal of energy intake. As mentioned previously, percent of calorie intake from alcohol was excluded from the last component of this indices and only percent of calorie intake from solid fats and added sugars was calculated.

### 2.4. Physical Activity

Physical activity was evaluated, using the adolescent modifiable activity questionnaire (MAQ), which includes the frequency and duration of common daily activities over the past year. The physical activity level of each person was expressed based on metabolic equivalent hours per week (METs h/week). One metabolic equivalents is the amount of oxygen consumed while sitting at rest and is equal to 3.5 mL O_2_ per kg body weight in a minute; subjects were then classified in 3 groups; sedentary (3 > MET), moderate (6 ≥ MET ≥ 3) and vigorous (6 < MET) physical activity [26].

### 2.5. Clinical and Biological Measurement

Weight was measured with subjects minimally clothed and without shoes, using digital scales and was recorded to the nearest 100 g. Height was measured, using a tape measure fixed to a wall, while subjects were in standing position, without shoes with their shoulders in a normally aligned and their head in the Frankfort horizontal plane, and was recorded to the nearest 0.5 cm. WC was measured at the umbilical level over light clothing, without any pressure to body surface, using an unstretched tape measure and was recorded to the nearest 0.1 cm [17].

After 12 to 14 h of fasting overnight, a fasting blood sample was drawn from all participants for measurement of glucose and lipid concentrations and centrifuged within 30 to 45 min. Fasting blood glucose (FBS) was measured on the day of blood collection by enzymatic colorimetric method using glucose oxidase. Triglyceride (TG) concentration was measured using commercially available enzymatic reagent (Pars Azemoon, Tehran, Iran) by the enzymatic colorimetric tests and with glycerol phosphate oxidase. High-density lipoprotein-cholesterol (HDL-C) was measured after precipitation of the apolipoprotein B-containing lipoproteins with phosphotungstic acid. Inter- and intra-assay coefficients of variation were 1.6% and 0.6%, respectively, for TG and 2% and 0.5% for HDL-C [17].

To measure blood pressure, participants were first asked to rest for 15 min. Then, while the subjects were in a seated position, a qualified physician measured blood pressure twice, using a standard mercury sphygmomanometer, after one initial measurement to determine peak inflation level; there was at least a 30 s interval between these two separate measurements, and the mean of 2 measurements was defined as the participant’s blood pressure. Systolic blood pressure (SBP) was defined as the appearance of the first sound (korotkoff phase 1), and diastolic blood pressure (DBP) was defined as the disappearance of the sound (korotkoff phase 5) during deflation of the cuff at a 2- to 3-mm/s decrement rate of the mercury column [27].

### 2.6. Definitions

According to the centers of disease control and prevention (CDC) cut-offs [28] and based on Iranian body mass index (BMI) percentiles [29], overweight (≥85th and <95th percentiles) and general obesity (≥95th percentile) were defined. The de Ferranti definition of metabolic syndrome (MetS) was the best predictor for MetS during early adulthood in our adolescent population [30] and was characterized as the presence of more than 3 of the following factors (1) fasting triglycerides ≥ 1.1 mmol/L (100 mg/dL); (2) HDL < 1.3 mmol/L (50 mg/dL), except in boys aged, 15 to 19 years, in whom the cutpoint was <1.2 mmol/L (45 mg/dL); (3) fasting glucose ≥ 6.1 mmol/L (110 mg/dL); (4) WC > 75th percentile for age and gender; and (5) systolic blood pressure > 90th percentile for gender, age, and height; since high WC is the most common MetS risk factors in Iranian adolescents [31], central obesity was defined according to the de Ferranti definition, based on Iranian WC percentiles [29]. In addition, MHO was established when subjects with general obesity had <2 abnormal cardio metabolic risk factors (elevated blood pressure, triglycerides, glucose, low HDL-C). Other obese participants were defined as MUO [8].

### 2.7. Statistical Analysis

All statistical analysis was conducted using the statistical package for social sciences (version 20.0; SPSS Inc., IBM, New York, NY, USA). All variables were shown to have a normal distribution. Diet quality scores were divided into quartile categories. To compare the characteristics and dietary intakes of participants across quartile categories of diet quality indices, analysis of covariance was used for continuous variables and chi-square test was used for dichotomous variables. Analysis of covariance preformed to assess mean values of BMI and WC across diet quality indices quartiles, adjusted for sex, age, energy intake and physical activity. To evaluate the relationship between these diet quality indices and risk of obesity, odds ratios (ORs) were calculated using logistic regression in three models, i.e., model I: adjustment for age and sex; model II: adjustment for age, sex and energy intake and model III: adjustment for age, sex, energy intake and physical activity. The lowest quartile category of indices was designated as the reference category. To determine *P*_trend_ within quartile categories of indices, linear regression coefficient was used for continuous variables and logistic regression was used for dichotomous variables. *p* values < 0.05 were considered statistically significant.

### 2.8. Ethical Standards

Written informed consent was obtained from all those aged ≥18 years and from the parents of those aged <18 years; the study protocol was approved (No. 707) by the ethics committee and research council of the Research Institute for Endocrine Science, Shahid Beheshti University of Medical Sciences.

## 3. Results

Of 722 participants, 45.7% were boys and 54.3% were girls, mean age 14.5 ± 2.9 and 14.9 ± 2.9 years, respectively. There was no significant difference in percentage of adolescents with central obesity, overweight, general obesity and MUO between genders. Only the prevalence of MHO was significantly higher in girls (Table 1).

Table 2 shows percentage of unhealthy metabolic factors in obese adolescents. MUO individuals have consistently higher metabolically unhealthy risk factors.

The mean score of DGAI, HEI-2005 and HEI-2010 in the present study was 9.9 ± 1.9 (ranged 4.50–16.25), 69.6 ± 8.7 (ranged 41.1–91.0) and 71.8 ± 9.1 (ranged 44.2–92.2), respectively. Table 3 shows the participant characteristics according to quartile categories of diet quality indices, those in the highest quartile category of DGAI score were significantly more likely to be girls than boys (32% vs. 22%, *p* < 0.001). Based on HEI-2005, older participants were more likely to obtain higher diet quality scores. After adjustment for sex and age, those in the highest quartile category of HEI-2010 were found to be more physically active than those in the lowest. There was no significant difference between other characteristics of participants and diet quality scores.

The dietary intakes of participants across quartile categories of diet quality indices are given in Table 4. After adjustment for sex, age and energy intake, the percent of energy intake from total fat, trans fatty acids, saturated fatty acid, cholesterol and sodium intake dropped significantly moving from the first to the last quartile of diet quality indices, except for HEI-2005 which showed no significant change with cholesterol intake. The percent of energy intake from carbohydrate and protein, fruit, vegetable and meat intakes increased significantly throughout the quartiles of diet quality indices. However, there were no significant differences between the highest and the lowest quartile category of DGAI in grain and dairy product intakes, the highest and the lowest quartile category of HEI-2005 in whole grain and dairy product intakes and the highest and the lowest quartile category of HEI-2010 in total fiber and grain intakes.

Adjusted mean values of BMI, WC and cardio metabolic risk factors across quartile categories of diet quality indices are given in Table 5. After adjustment for sex, age, energy intake and physical activity, mean values of BMI and WC showed a significant decreasing trend, according to quartiles of HEI-2010. Also, there was a direct association between DGAI and HEI-2005 scores and HDL-C concentration; and an inverse association between HEI-2005 score and fasting blood glucose and triglyceride concentrations. No significant relationship was observed between BMI, WC and other cardio metabolic risk factors and indices scores.

Table 6 shows ORs for different types of obesity (including central obesity, general obesity, metabolically healthy obesity and metabolically un-healthy obesity) as dichotomous variables. After adjustment for age, sex, energy intake and physical activity, being in the highest quartile category of HEI-2010 reduced the risk of central obesity by 37% (OR: 0.63; 95% CI 0.44–0.95; *P*_trend_ = 0.04) and general obesity by 38% (OR: 0.62; 95% CI 0.38–0.93; *P*_trend_ = 0.03). No significant difference was observed in odds of different types of obesity according the quartile categories of DGAI and HEI-2005 scores.

## 4. Discussion

In the current study, we compared the association of DGAI, HEI-2005 and HEI-2010 with risk of different types of obesity. Our findings indicate that participants who had high adherence with HEI-2010 had a lower risk of general and central obesity. Considering the limited research available, examining the relationship between diet quality indices and obesity in adolescents, the present study provides useful information for establishing more accurate dietary guidelines in our country.

Compared with American children and adolescents, Iranian adolescents have higher diet quality (based on HEI-2005: 55.9 vs. 69.6 and based on HEI-2010: 49.8 vs. 71.8) [32]. It is worth noting that the prevalence of obesity (BMI ≥ 95th percentile of the BMI-for-age) in Iranian adolescents was less than that of American children and adolescents (15.2 vs. 16.9) [6].

Regarding the impact of dietary intakes on obesity, several studies have investigated the relationship between diet quality indices and obesity in children and adolescents. Linardakis and colleagues have shown that BMI decreased with increase in HEI-1995 scores (*P*_trend_ = 0.04), whereas no significant relationship was observed for WC [14]. According to Jennings and colleagues, in 9 to 10 years old British children, higher Diet Quality Index (DQI) and Healthy Diet Indicator (HDI) scores were associated with lower WC; DQI was also associated with lower BMI; however, there was no significant relationship between MDS and weight status [33]. Likewise, another study also reported no association between MDS and risk of high WC in 12 to 17 years adolescents [15]. Also, compared with metabolically abnormal obesity, metabolically healthy obese adolescents had higher HEI-2005 scores [8]. In Iranian adolescents, there were no significant associations between HEI-1995 score and BMI or abdominal obesity. Also, those in the highest tertile of mean adequacy ratio had higher BMI and WC [34].

Findings from studies in adults are also contradictory. An inverse association was observed between HEI-1995 and risk of overweight, obesity and abdominal obesity in the third National Health and Nutrition Examination Survey (NHANES III) [35,36]. In addition, it has been shown that HEI-1995, Recommended Foods Score (RFS-24 hour recall) and Dietary Diversity Score were negative predictors of BMI [37]. Also, Nurses’ findings of the Health Study (NHS) suggest that individuals with closer adherence to the HEI-2005 tended to have lower BMI [38]. However, in Iranian adults, adherence to HEI-2005, MDS, and DQI could not predict BMI and WC after 6.7 years of follow-up [39]. In a cross-sectional analysis of Americans, the DGAI score was inversely related to WC [12], whereas, in Iranian adults, this relationship has not been observed [40]. Furthermore, in a prospective study DGAI score predicted obesity risk in men, but not in women [41].

Based on our findings, indices can represent diet quality of adolescents to some extent, such that by increasing diet quality score, percent of calorie intake from fat, saturated fatty acids and trans fatty acids decreased and intakes of fruits and vegetables and meat increased, indicating partially the validity of these three indices in our population.

Among the indices examined in this study, only HEI-2010 had an inverse relationship with the risk of general and central obesity; BMI and WC decreased significantly across the quartiles of this index. Generally, it is accepted that obesity is the result of positive energy balance [42]; the ability of diet quality indices in predicting general and central obesity depends on how these indices control changes in energy balance, and also on the structure and method of scoring in each index [37]. HEI-2005 and HEI-2010 are based on an energy density approach that focuses on intakes of food groups and nutrients per 1000 kcal energy intake [24,25]. Although this approach results in balance among food groups intakes, it does not consider each individual’s needs for energy and extra energy consumption. In other words, these indices do not allocate negative scores to intakes of energy above the energy requirement. However, it should be noted that scoring for various components of food has changed in HEI-2010, compared to HEI-2005, e.g., if a person consumes whole grains >1.5 ounce per 1000 kcal per day, based on HEI-2005 and HEI-2010 he/she gets a score of 5 and 10, respectively [24,25]. Also, in HEI-2005, if total grain intake was >3 ounces per 1000 kcal per day, a score of 5 is given; however, in HEI-2010, if refined grains intake was <1.8 ounce per 1000 kcal per day, the person is scored 10 [24,25], indicating that HEI-2010 places more emphasis on reducing consumption of refined grains and increasing consumption of whole grains. It should be noted that previous studies have shown that the main source of energy in Iranians was grains (especially the refined type) [43]. Moreover, HEI-2010 considers the ratio of “(PUFA + MUFA)/SFA” as a one item scored 10, instead of 2 items (the amount of oil and saturated fatty acid consumed) that their scores of which are 20 [25]. The difference in association between these two indices and obesity status may be attributable to differences in the maximum scores of the components and it seems that energy-rich food sources (like whole grains, refined grains and fats) have been scored better in HEI-2010.

In DGAI, food group intakes recommendations are based on energy level requirements [23]. Items of this index have a maximum value of 1.0. For four food groups that are considered energy dense (starchy vegetables, meat and beans, cereals and milk and dairy products), this index imposed an overconsumption penalty for those who exceeded the recommended intake by ≥0.5 servings by assigning only 0.5 points [23], indicating that if a person consumes more than the amount required from these for four food groups, consuming hence excess energy, of a total 20 points of this index, he/she gets a maximum 2 points as a penalty. Therefore, it seems that DGAI does not consider extra energy intake in calculating the diet quality score.

According to our findings in all diet quality indices higher diet quality scores are associated with lower energy density. Due to differences in genetic predisposition, environmental factors and specific characteristics of dietary pattern in different ethnicities, it is better that diet quality indices be revised in each population for better match with the health targets [40].

To address the several potential limitations of this research, first, the cross-sectional design of the present study cannot demonstrate causal relations and only generates hypotheses regarding diet quality and weight status. Secondly, an important limitation to consider when interpreting our results was the use of FFQ for collecting the dietary data; despite its common use for characterizing habitual intakes, its weakness in the quantification of nutrient intake is well-recognized. However, being easy to complete and analyze, FFQs are the primary source for data collection in large epidemiologic surveys, being more informative of habitual intake than data on intake on a few specific days [44]. In addition, analysis of the data for both sexes was not possible due to the low sample size. Moreover, because the Iranian FCT is incomplete and provides limited data on nutrient content of raw foods and beverages, the American FCT was used for analyzing an Iranian diet. Since there are no standard dietary guidelines accessible for Iranian populations, the diet quality of adolescents was assessed based on DGA-2005 and DGA-2010, which have been developed for American populations.

The population-based analysis and conducting of the study in a developing country under nutrition transition are the main strengths of the present study. In addition, measurement and control of many known confounders can also be considered strengths of our study.

## 5. Conclusions

To our knowledge, this was the first study that compared three diet quality indices (DGAI, HEI-2005 and HEI-2010) in relation to risk of different types of obesity in adolescents. Findings of the current study demonstrate that only adherence to the HEI-2010 was associated with reduced odds of general obesity and central obesity in a sample of Iranian adolescents, whereas the other two indices (DGAI and HEI-2005) had no significant relationship with obesity status. Further studies including dietary intervention studies are needed to confirm the effect of HEI-2010 on the intermediate markers of disease.

## Figures and Tables

**Table 1:** Percentage of adolescents with overweight, general obesity and central obesity in the Tehran Lipid and Glucose Study.

Phenotypes of Obesity	Girls	Boys	Total	*p* Value ^c^
	(*n* = 392)	(*n* = 330)	(*n* = 722)	
Central obesity ^b^ (%)	50.0	54.2	51.9	0.26
Overweight ^a^ (%)	21.2	20.9	21.1	0.12
General obesity ^a^ (%)	12.8	18.2	15.2	
MHO ^d^ (%)	8.8	5.4	6.9	0.04
MUO ^e^ (%)	9.4	7.4	8.3	0.20

^a^ According to the Centers of Disease Control and Prevention (CDC) cut-offs and based on Iranian percentiles; ^b^ Central obesity was defined according to the de Ferranti definition and based on Iranian percentiles; ^c^ *p* value determined using Chi-square; ^d^ metabolically healthy obesity; ^e^ metabolically unhealthy obesity.

**Table 2:** Percentage of unhealthy metabolic factors in obese adolescents in the Tehran Lipid and Glucose Study ^a^.

Unhealthy Metabolic Factors	General Obesity	Central Obesity	MHO ^b^	MUO ^c^	Total
	(*n* = 110)	(*n* = 375)	(*n* = 50)	(*n* = 60)	(*n* = 722)
Hyperglycemia	1.8 (2)	2.1 (8)	0.0 (0)	3.3 (2)	1.4 (10)
Hypertriglyceridemia	60.0 (66)	48.3 (181)	24.0 (12)	90.0 (54)	35.9 (259)
Low HDL-C	68.2 (75)	58.4 (219)	34.0 (17)	96.7 (58)	47.0 (339)
Hypertension	21.8 (24)	15.5 (58)	4.0 (2)	36.7 (22)	11.8 (85)

^a^ Percentage (number), percentage determined using chi-square; ^b^ metabolically healthy obese; ^c^ metabolically unhealthy obese.

**Table 3:** Participant characteristics according to quartile categories of diet quality indices score in the Tehran Lipid and Glucose Study adolescents ^a^.

Characteristics	Quartiles of DGAI Score				*P*_trend_^b^	Quartiles of HEI-2005 Score				*P*_trend_	Quartiles of HEI-2010 Score				*P*_trend_
	1	2	3	4		1	2	3	4		1	2	3	4	
	(4.5–8.5)	(8.75–10.0)	(10.25–11.25)	(11.5–16.25)		(41.3–64.1)	(64.2–70.4)	(70.5–76.0)	(76.1–91.1)		(44.2–65.6)	(65.7–73.1)	(73.2–78.6)	(78.7–92.9)	
Participants (no)	200	200	150	172		188	179	178	177		192	176	178	176	
Girls (%)	22.2	25.0	20.9	31.9	<0.001	24.5	24.2	25.8	25.5	0.64	23.7	24.5	24.7	27.0	0.16
Age (years)	14.8	14.9	14.6	14.7	0.73	14.3	14.8	14.7	15.2	0.006	14.6	14.6	14.6	15.1	0.39
Central obesity (%)	57.0	51.5	51.3	47.1	0.29	55.9	54.2	47.8	49.7	0.37	57.3	52.8	49.4	47.7	0.26
Overweight (%)	18.5	22.0	21.3	22.7	0.27 ^c^	16.0	25.7	19.7	23.2	0.07	19.3	22.7	19.7	22.7	0.81
General obesity (%)	20.0	10.5	15.3	15.1		20.3	14.5	10.1	15.8		17.2	15.9	15.7	11.9	
MHO (%)	9.0	6.0	6.0	6.4	0.59	8.5	8.9	3.4	6.8	0.14	8.9	8.0	5.1	5.7	0.42
MUO (%)	11.0	4.5	9.3	8.7	0.11	11.7	5.6	6.7	9.0	0.15	8.3	7.9	10.6	6.2	0.51
Physical activity score ^d^ (MET) ^e^, mean	7.58	7.19	5.53	5.88	0.12	5.15	7.15	6.33	8.02	0.10	4.13	6.07	9.41	7.15	0.04

^a^ DGAI score ranged from 0 to 19, and HEI-2005 and HEI-2010 ranged from 0 to 100 points; ^b^ *P*_trend_ < 0.05, using linear regression for continuous variables and Chi-square for dichotomous variables; ^c^ *p* value for overweight and general obesity determined using Chi-square; ^d^ Physical activity score adjusted for sex and age; ^e^ Metabolic Equivalents.

**Table 4:** Mean (SE) of daily nutrient intakes across the quartile categories of diet quality index scores in Tehran Lipid and Glucose Study adolescents ^a^.

Dietary Intakes/Day	Quartiles of DGAI Score				*P*_trend_^b^	Quartiles of HEI-2005 Score				*P*_trend_^b^	Quartiles of HEI-2010 Score				*P*_trend_^b^
	1	2	3	4		1	2	3	4		1	2	3	4	
Total reported energy (kcal)	2656 (58)	2657 (58)	2712 (67)	2887 (63)	0.002	2716 (60)	2779 (61)	2688 (62)	2709 (62)	0.98	2606 (59)	2718 (62)	2767 (61)	2811 (62)	0.02
Energy density (kcal/g)	1.24 (0.15)	1.18 (0.15)	1.09 (0.17)	1.06 (0.16)	<0.001	1.28 (0.15)	1.17 (0.15)	1.09 (0.15)	1.05 (0.15)	<0.001	1.24 (0.15)	1.17 (0.16)	1.13 (0.16)	1.05 (0.16)	<0.001
Carbohydrate (% of total energy)	52.7 (0.4)	55.2 (0.4)	57.5 (0.4)	58.0 (0.4)	<0.001	53.0 (0.4)	55.4 (0.4)	57.2 (0.4)	57.2 (0.4)	<0.001	54.9 (0.4)	55.0 (0.4)	56.1 (0.4)	56.7 (0.4)	0.004
Protein (% of total energy)	12.9 (0.2)	13.4 (0.2)	13.6 (0.2)	14.6 (0.2)	<0.001	12.6 (0.2)	13.4 (0.2)	13.7 (0.2)	14.7 (0.2)	<0.001	12.6 (0.2)	13.4 (0.2)	13.8 (0.2)	14.7 (0.2)	<0.001
Fat (% of total energy)	34.1 (0.4)	31.3 (0.4)	28.8 (0.4)	27.6 (0.4)	<0.001	34.0 (0.4)	31.2 (0.4)	29.2 (0.4)	28.0 (0.4)	<0.001	31.4 (0.4)	31.9 (0.4)	29.9 (0.4)	29.5 (0.4)	<0.001
Trans (% of total energy)	1.68 (0.05)	1.46 (0.05)	1.16 (0.06)	0.88 (0.06)	<0.001	1.86 (0.05)	1.34 (0.05)	1.16 (0.06)	0.88 (0.06)	<0.001	1.56 (0.06)	1.36 (0.06)	1.25 (0.06)	1.08 (0.06)	<0.001
Saturated fat (% of total energy)	11.6 (0.2)	10.3 (0.2)	9.5 (0.2)	9.1 (0.2)	<0.001	11.9 (0.2)	10.8 (0.2)	9.3 (0.2)	9.2 (0.2)	<0.001	10.7 (0.2)	11.1 (0.2)	10.0 (0.2)	9.6 (0.2)	<0.001
Cholesterol (mg)	281 (8)	250 (8)	237 (9)	241 (9)	<0.001	255 (8)	260 (8)	239 (8)	262 (8)	0.63	247 (8)	254 (8)	249 (8)	266 (8)	0.06
Sodium (mg)	3930 (71)	3928 (71)	3799 (81)	3780 (78)	0.02	4246 (69)	4033 (71)	3794 (71)	3367 (71)	<0.001	4264 (69)	3974 (71)	3798 (71)	3394 (72)	<0.001
Total fiber (g)	40.8 (1.1)	46.5 (1.1)	48.6 (1.3)	54.7 (1.2)	<0.001	45.4 (1.2)	45.8 (1.2)	48.7 (1.2)	49.5 (1.2)	0.002	49.2 (1.1)	44.7 (1.1)	46.4 (1.3)	48.8 (1.2)	0.40
Grain (ozeq/day)	10.9 (0.3)	11.9 (0.3)	12.1 (0.4)	11.3 (0.4)	0.31	12.5 (0.3)	11.6 (0.3)	11.6 (0.3)	10.3 (0.3)	0.001	12.8 (0.3)	11.5 (0.3)	11.6 (0.3)	10.1 (0.3)	<0.001
Whole grain (ozeq/day)	4.35 (0.31)	5.12 (0.31)	6.28 (0.35)	5.97 (0.34)	<0.001	5.56 (0.31)	5.44 (0.32)	5.77 (0.33)	4.60 (0.33)	0.73	4.19 (0.31)	5.41 (0.33)	6.27 (0.33)	5.62 (0.33)	<0.001
Fruit (cup eq/day)	1.91 (0.16)	2.49 (0.16)	3.27 (0.19)	3.78 (0.18)	<0.001	1.57 (0.17)	2.59 (0.17)	3.42 (0.17)	3.70 (0.17)	<0.001	1.58 (0.16)	2.71 (0.17)	3.22 (0.17)	3.80 (0.17)	<0.001
Vegetables ^c^ (cup eq/day)	2.56 (0.10)	3.00 (0.10)	3.11 (0.11)	3.69 (0.10)	<0.001	2.67 (0.10)	2.92 (0.10)	3.09 (0.10)	3.60 (0.10)	<0.001	2.59 (0.10)	2.92 (0.10)	3.14 (0.10)	3.66 (0.10)	<0.001
Meat ^d^ (ozeq/day)	3.80 (0.13)	4.10 (0.13)	4.11 (0.15)	4.94 (0.15)	<0.001	3.30 (0.13)	3.87 (0.13)	4.34 (0.13)	5.43 (0.13)	<0.001	3.45 (0.13)	3.90 (0.14)	4.45 (0.14)	5.15 (0.14)	<0.001
Dairy products (cup eq/day)	2.67 (0.09)	2.20 (0.09)	2.27 (0.10)	2.32 (0.10)	0.07	2.37 (0.10)	2.54 (0.10)	2.14 (0.10)	2.44 (0.10)	0.67	2.32 (0.10)	2.40 (0.10)	2.28 (0.10)	2.50 (0.10)	0.02

^a^ Values determined using ANCOVA after adjustment for age, sex and energy intake; ^b^ *P* for trend determined using linear regression; ^c^ Including vegetables and legumes; ^d^ According to the dietary guidelines for Americans recommendations, legumes were assigned to the meat group for those who had not achieved maximum points of meats, and if the meat point was maximized an extra amount was counted in the vegetable group.

**Table 5:** Mean (SE) for anthropometric measures and cardio metabolic risk factors across the quartile categories of diet quality indices score in Tehran Lipid and Glucose Study adolescents ^a^.

Anthropometric Measures	Quartiles of DGAI Score				*P*_trend_^b^	Quartiles of HEI-2005 Score				*P*_trend_^b^	Quartiles of HEI-2010 Score				*P*_trend_^b^
	1	2	3	4		1	2	3	4		1	2	3	4	
BMI (kg/m^2^)	22.3 (0.3)	21.5 (0.3)	21.9 (0.3)	21.9 (0.3)	0.23	22.3 (0.3)	22.1 (0.3)	21.3 (0.3)	22.0 (0.3)	0.38	22.2 (0.3)	22.1 (0.3)	21.9 (0.3)	21.5 (0.3)	0.03
Waist circumference (cm)	78.0 (0.8)	75.4 (0.8)	76.8 (0.9)	76.5 (0.9)	0.20	77.3 (0.8)	77.6 (0.8)	75.4 (0.8)	76.3 (0.9)	0.32	77.2 (0.8)	77.3 (0.9)	76.6 (0.9)	75.4 (0.9)	0.04
Fasting blood glucose (mg/dL)	92.3 (0.5)	92.4 (0.5)	92.6 (0.6)	91.5 (0.6)	0.30	92.9 (0.6)	92.4 (0.6)	91.9 (0.6)	91.6 (0.6)	0.04	92.5 (0.5)	92.9 (0.5)	91.9 (0.6)	91.6 (0.6)	0.07
Triglycerides (mg/dL)	100.1 (3.4)	96.6 (3.4)	92.3 (4.0)	95.9 (3.8)	0.28	103.7 (3.5)	97.2 (3.6)	91.8 (3.6)	92.9 (3.6)	0.04	100.7 (3.5)	97.1 (3.6)	94.6 (3.8)	93.3 (3.7)	0.06
HDL-C (mg/dL)	48.7 (0.7)	50.0 (0.7)	50.4 (0.8)	50.6 (0.8)	0.03	48.8 (0.7)	49.6 (0.8)	50.3 (0.8)	51.0 (0.8)	0.01	49.6 (0.7)	49.3 (0.8)	49.8 (0.8)	50.8 (0.8)	0.27
Systolic blood pressure (mmHg)	102.7 (0.8)	100.6 (0.8)	100.0 (0.9)	101.9 (0.9)	0.25	101.9 (0.9)	101.7 (0.9)	100.8 (0.9)	100.0 (0.9)	0.68	101.3 (0.8)	101.8 (0.9)	101.4 (0.9)	101.0 (0.9)	0.79
Diastolic blood pressure (mmHg)	68.2 (0.6)	66.4 (0.6)	66.1 (0.7)	67.5 (0.7)	0.13	67.4 (0.7)	67.4 (0.7)	66.4 (0.7)	67.2 (0.7)	0.46	67.8 (0.7)	66.7 (0.7)	67.7 (0.7)	66.2 (0.7)	0.96

^a^ Values determined using ANCOVA adjusted for age, sex, energy intakes and physical activity; ^b^ *P* for trend determined using linear regression.

**Table 6:** Odds ratio (95% CI) for central obesity, general obesity, metabolically healthy obesity and metabolically un-healthy obesity across quartile categories of diet quality indices score in Tehran Lipid and Glucose Study adolescents *.

Obesity Phenotypes		Quartiles of DGAI Score				*P*_trend_^†^	Quartiles of HEI-2005 Score				*P*_trend_^†^	Quartiles of HEI-2010 Score				*P*_trend_^†^
		1	2	3	4		1	2	3	4		1	2	3	4	
General obesity	Model I ^‡^	1	0.47 (0.26–0.84)	0.76 (0.43–1.34)	0.81 (0.46–1.41)	0.53	1	0.66 (0.38–1.14)	0.44 (0.24–0.82)	0.72 (0.42–1.25)	0.10	1	0.93 (0.57–1.33)	0.91 (0.52–1.11)	0.66 (0.37–0.99)	0.06
	Model II ^§^	1	0.45 (0.26–0.84)	0.75 (0.42–1.33)	0.77 (0.44–1.36)	0.44	1	0.65 (0.37–1.13)	0.44 (0.24–0.81)	0.72 (0.42–1.25)	0.10	1	0.91 (0.54–1.31)	0.89 (0.50–1.10)	0.64 (0.35–0.96)	0.05
	Model III ^׀^	1	0.47 (0.26–0.84)	0.76 (0.43–1.34)	0.78 (0.45–1.38)	0.47	1	0.64 (0.36–1.11)	0.43 (0.23–0.80)	0.70 (0.41–1.21)	0.09	1	0.89 (0.51–1.29)	0.85 (0.48–1.08)	0.62 (0.34–0.93)	0.03
Central obesity	Model I	1	0.81 (0.54–1.20)	0.79 (0.51–1.21)	0.68 (0.45–1.04)	0.08	1	0.96 (0.63–1.45)	0.74 (0.49–1.12)	0.83 (0.54–1.25)	0.21	1	0.84 (0.55–1.27)	0.73 (0.48–1.10)	0.71 (0.54–1.25)	0.07
	Model II	1	0.81 (0.54–1.20)	0.79 (0.51–1.21)	0.68 (0.44–1.03)	0.07	1	0.96 (0.63–1.45)	0.84 (0.49–1.12)	0.83 (0.54–1.26)	0.21	1	0.84 (0.55–1.26)	0.73 (0.48–1.10)	0.70 (0.46–1.07)	0.07
	Model III	1	0.81 (0.54–1.20)	0.80 (0.52–1.23)	0.68 (0.45–1.05)	0.08	1	0.95 (0.63–1.43)	0.74 (0.49–1.11)	0.81 (0.54–1.23)	0.19	1	0.82 (0.54–1.27)	0.70 (0.46–1.07)	0.63 (0.44–0.95)	0.04
MHO	Model I	1	0.65 (0.30–1.39)	0.69 (0.29–1.59)	0.81 (0.36–1.81)	0.53	1	1.02 (0.49–2.12)	0.36 (0.13–0.96)	0.73 (0.33–1.61)	0.15	1	0.90 (0.43–1.90)	0.55 (0.24–1.22)	0.62 (0.27–1.41)	0.15
	Model II	1	0.65 (0.30–1.39)	0.66 (0.28–1.53)	0.75 (0.33–1.69)	0.42	1	1.01 (0.48–2.10)	0.36 (0.14–0.96)	0.73 (0.33–1.62)	0.16	1	0.88 (0.41–1.85)	0.53 (0.22–1.23)	0.58 (0.25–1.32)	0.11
	Model III	1	0.64 (0.30–1.39)	0.66 (0.28–1.53)	0.75 (0.33–1.68)	0.42	1	1.01 (0.48–2.12)	0.36 (0.14–0.97)	0.73 (0.33–1.63)	0.16	1	0.87 (0.41–1.82)	0.52 (0.22–1.23)	0.57 (0.25–1.32)	0.11
MUO	Model I	1	0.35 (0.17–0.86)	0.83 (0.42–1.74)	0.85 (0.41–1.70)	0.79	1	0.44 (0.20–0.97)	0.55 (0.26–1.15)	0.76 (0.38–1.50)	0.37	1	0.96 (0.45–2.04)	1.33 (0.66–2.68)	0.75 (0.33–1.68)	0.81
	Model II	1	0.38 (0.17–0.86)	0.85 (0.42–1.74)	0.83 (0.40–1.69)	0.77	1	0.44 (0.20–0.97)	0.55 (0.26–1.15)	0.76 (0.38–1.50)	0.37	1	0.96 (0.45–2.03)	1.32 (0.65–2.67)	0.74 (0.33–1.67)	0.79
	Model III	1	0.38 (0.17–0.86)	0.88 (0.43–1.79)	0.85 (0.41–1.75)	0.84	1	0.42 (0.19–0.94)	0.53 (0.25–1.12)	0.72 (0.36–1.44)	0.32	1	0.93 (0.44–1.99)	1.23 (0.60–2.52)	0.71 (0.32–1.60)	0.66

* Values determined using logistic regression; ^†^ *P* for trend determined using logistic regression; ^‡^ Adjustment for age, sex; ^§^ Adjustment for age, sex and energy intakes; ^׀^ Adjustment for age, sex, energy intakes and physical activity.
